# TRI microparticles prevent inflammatory arthritis in a collagen-induced arthritis model

**DOI:** 10.1371/journal.pone.0239396

**Published:** 2020-09-23

**Authors:** Ethan J. Bassin, Abigail R. Buckley, Jon D. Piganelli, Steven R. Little

**Affiliations:** 1 Department of Immunology, University of Pittsburgh, Pittsburgh, Pennsylvania, United States of America; 2 Division of Pediatric Surgery, Department of Surgery, Children’s Hospital of Pittsburgh, University of Pittsburgh, Pittsburgh, Pennsylvania, United States of America; 3 Department of Chemical Engineering, University of Pittsburgh, Pittsburgh, Pennsylvania, United States of America; 4 Department of Bioengineering, University of Pittsburgh, Pittsburgh, Pennsylvania, United States of America; 5 Department of Ophthalmology, University of Pittsburgh, Pittsburgh, Pennsylvania, United States of America; 6 Department of Pharmaceutical Science, University of Pittsburgh, Pittsburgh, Pennsylvania, United States of America; Hanyang University, REPUBLIC OF KOREA

## Abstract

Despite recent progress in the treatment of rheumatoid arthritis (RA), many patients still fail to achieve remission or low disease activity. An imbalance between auto-reactive effector T cells (Teff) and regulatory T cells (Treg) may contribute to joint inflammation and damage in RA. Therefore, restoring this balance is a promising approach for the treatment of inflammatory arthritis. Accordingly, our group has previously shown that the combination of TGF-β-releasing microparticles (MP), rapamycin-releasing MP, and IL-2-releasing MP (TRI MP) can effectively increase the ratio of Tregs to Teff *in vivo* and provide disease protection in several preclinical models. In this study TRI MP was evaluated in the collagen-induced arthritis (CIA) model. Although this formulation has been tested previously in models of destructive inflammation and transplantation, this is the first model of autoimmunity for which this therapy has been applied. In this context, TRI MP effectively reduced arthritis incidence, the severity of arthritis scores, and bone erosion. The proposed mechanism of action includes not only reducing CD4^+^ T cell proliferation, but also expanding a regulatory population in the periphery soon after TRI MP administration. These changes were reflected in the CD4^+^ T cell population that infiltrated the paws at the onset of arthritis and were associated with a reduction of immune infiltrate and inflammatory myeloid cells in the paws. TRI MP administration also reduced the titer of collagen antibodies, however the contribution of this reduced titer to disease protection remains uncertain since there was no correlation between collagen antibody titer and arthritis score.

## Introduction

Rheumatoid arthritis (RA) is an autoimmune disease of chronic joint inflammation affecting 0.5–1% of the population in Western countries, and approximately 1.5 million people in the U.S. [1,2]. RA joint inflammation leads to irreversible damage to cartilage and bone. This can be debilitating for patients and the corresponding decreased work capacity is the main driver of the estimated $46 billion societal burden of RA in the U.S. [3]. Tremendous progress has been made over the past few decades in optimizing treatment with conventional synthetic disease-modifying antirheumatic drugs (DMARDs), developing biologic DMARDs including TNF-α inhibitors, and the recent introduction of Janus kinase (JAK) inhibitors. However, none of these therapies have been able to achieve low disease activity in even 50% of methotrexate-naive patients, and with each line of further therapy there is a diminishing return of patients who adequately respond [4,5]. It has been suggested that a similar maximum efficacy has been observed across RA drug types because regardless of the direct target, all of these drugs ultimately act by blocking TNF-α and/or IL-6 [6]. Thus, a substantial population of RA patients remain underserved by existing treatments, and there is a need to develop new treatments with a different mechanism of action.

Although there is not a natural spontaneous animal model of RA, collagen-induced arthritis (CIA) is a widely used mouse model that has many similarities to RA. CIA resembles RA in some important histological and radiographic measures including fibrin deposition, synovial hyperplasia, mononuclear infiltration, and bone erosion [7–9]. While collagen II (CII) is the initiating antigen in CIA, the defining antibodies of seropositive RA—rheumatoid factor (antibodies to self IgG-Fc) and anti-citrullinated protein antibodies (ACPAs)—have been detected in CIA with the latter shown to contribute to disease pathogenesis [10,11]. Both auto-antibodies and T cells contribute to CIA pathogenesis. Anti-CII antibody administration is sufficient to transfer CIA [12], assuming it is of appropriate dose, avidity, and isotype [10]. CD4^+^ T cells play an important role in generation of anti-CII antibodies in CIA [13–15], and CII or citrullinated protein specific CD4^+^ T cells can also exacerbate disease by trafficking to the joints and producing inflammatory cytokines [10,12,16].Together, the complement activation by auto-antibodies and CD4^+^ T cell production of IFN-γ and/or IL-17 is thought to lead to recruitment and activation of innate immune cells which in turn produce TNF-α and IL-1β leading to tissue swelling and destruction [10,17,18].

The balance between regulatory T cells (Tregs) and auto-reactive effector T cells (Teff) influences arthritis disease progression in both RA and CIA. Tregs restrain Teff from causing damage to healthy tissue in the elimination of pathogens as well as play a critical role in peripheral tolerance by preventing auto-reactive T cells from causing autoimmunity. Tregs have a variety of possible mechanisms to directly suppress Teff or indirectly suppress Teff through actions on antigen presenting cells (APCs). These mechanisms can either be contact dependent, such as expression of CTLA-4 or other co-inhibitory receptors, or contact independent such as the production of immunosuppressive cytokines or adenosine via CD39 and CD73 [19]. Canonical Tregs express the transcription factor FoxP3, and their importance in maintaining self-tolerance is illustrated by Foxp3 mutation which results in fatal multi-organ autoimmune disease in both mice (scurfy mice) and humans (IPEX syndrome) [20]. However, non-canonical FoxP3^-^ regulatory CD4^+^ T cells [21,22] and other regulatory populations [23–25] have also been identified in a variety of contexts. RA is associated with reduced suppressive ability of Tregs, due to a Treg intrinsic defect as well as the inflammatory milieu [26–28]. While in the CIA model, Treg depletion accelerates the onset of disease [29] and cell-therapy with collagen-specific Tregs can reverse disease progression [30].

A treatment capable of re-establishing Treg-Teff balance in RA may be able to restore tolerance and protect against disease progression. Polyclonal Treg cell-therapy is one approach to achieve this and while initial trials in several auto-immune and transplant indications have demonstrated safety, efficacy has not yet been proven [31,32]. There are substantial challenges to polyclonal Treg cell-therapy including the cost and complexity of good manufacturing practice (GMP) isolation and cell expansion [33], as well as concerns about potency [33,34], non-specific immunosuppression [35], and Treg instability or plasticity [36]. Approaches to restore Treg-Teff balance that use auto-antigen and/or localized immunomodulatory agents could avoid many of these issues. Several such approaches using citrullinated peptides [11,37], CII peptide-MHC II complex [38,39] or liposomes encapsulating antigen and NF-κB inhibitor [40] have demonstrated success in inflammatory arthritis models, but it remains unclear if these technologies will translate to RA. In particular, the immune response to antigen is highly dependent on antigen dose and context including cytokine milieu and prior exposure [41–43].

Previously, we reported the use of polymeric microparticles (MP) which release TGF-β, rapamycin, and IL-2 (TRI MP) [44] so that endogenous antigen can be presented in a tolerance-promoting local immunological microenvironment. This combination was initially chosen due to the role of each of these factors in promoting Treg induction and expansion [44–48]. IL-2 is needed for T cell differentiation/proliferation, and low doses expand Tregs [49]. In addition to TGF-β and rapamycin promoting Treg expansion and naïve T cell differentiation into Tregs (which is in part achieved by effects on APCs), these factor can also directly suppress Teff cell proliferation [50–52]. Subcutaneous TRI MP administration at the site of inflammation has previously demonstrated an ability to expand Tregs and limit Teff levels, resulting in disease prevention or therapeutic treatment in several preclinical models [53–55]. However, TRI MP has not been previously evaluated in a model of autoimmunity. These studies have also shown the combination of all three drugs is more effective than any drug alone or pair of two drugs, that TRI MP can confine drug activity to a local area resulting in antigen-specific immunosuppression, and that sustained release of drug from TRI MP is more potent than equivalent unencapsulated doses. Furthermore, injection of microparticles into inflamed joints could be a viable clinical approach as intra-articular injection of corticosteroid containing microparticles is already FDA-approved for osteoarthritis pain management [56].

Here we demonstrate the ability of TRI MP to prevent arthritic inflammation and bone erosion of the paws in a CIA model of arthritis. The proposed mechanism of this protective effect involves reduced T cell proliferation and the expansion of a regulatory cell population which together ultimately resulted in less immune infiltration of the paws. Anti-CII IgG antibodies were also reduced by TRI MP administration, but not found to contribute to the arthritis prevention provided by this treatment.

## Materials and methods

### Microparticle fabrication

TRI MP were fabricated using an emulsion-solvent evaporation method as previously described [54]. A 5% w/v polymer solution was prepared by dissolving 200 mg of Poly (lactic-co-glycolic) acid (PLGA) in 4 mL of dichloromethane (DCM) (Sigma Aldrich, St. Louis, MO). For IL-2 and Rapamycin, 200 mg of acid terminated PLGA (50:50 lactide:glycolide, MW:7,-17 kDa, Sigma Aldrich) was used for polymer MP. For TGF-β, 170 mg of ester terminated PLGA (50:50 lactide:glycolide, MW:7,000–17,000) (Sigma Aldrich) and 30 mg of mPEG-PLGA (50:50 lactide:glycolide, 5–20 kDa, PolySciTech, West Lafayette, IN) were used for polymer MP.

For TGF-β and IL-2, primary emulsions were formed by adding 5 μg of recombinant protein (hTGF-β from PeproTech, Rocky Hill, NJ) (mIL-2 from R&D Systems, Minneapolis, MN), dissolved in 200 μL of deionized (DI) water or phosphate buffered saline (PBS) respectively, to the organic polymer phase, and sonicating at 25% amplitude for 10 s (Active Motif, Carlsbad, CA). For Rapamycin, 1 mg of rapamycin (Alfa Aesar, Ward Hill, MA) dissolved in 100 μL of dimethyl sulfoxide was added to the polymer solution without sonication. Blank MP was made for each type of MP using vehicle control solution.

The resulting primary emulsion or polymer-drug solution was poured into 60 mL of 2% w/v poly(vinyl alcohol) (PVA, MW ~25 kDa, 98% hydrolyzed, Polysciences, Warrington, PA) in DI water (or 51.6 mM NaCl for IL-2) and homogenized (L4RT-1, Silverson, East Longmeadow MA) at 3,000 rpm for 1 min. The resulting double or single emulsion was then poured into 80 mL of 1% w/v PVA in DI water or (51.6 mM NaCl for IL-2) and stirred (600 rpm) for 3 h to allow DCM to evaporate. TGF-β and IL-2 emulsions were homogenized and stirred on ice. After stirring, MP were collected by centrifugation (200 g, 5 min, 4°C) and washed 4 times with DI water before lyophilizing for 48 hours.

### Microparticle characterization

MP surface morphology was characterized using scanning electron microscopy (JEOL, JSM-6330F, Peabody, MA), and the size distribution of microparticles was measured with a Beckman Coulter Counter (Multisizer-3, Beckman Coulter, Brea, CA).

Total drug loading of MP was assessed as previously described [53,57]. For TGF-β and IL-2, drug was extracted using DCM and PBS with 0.1% sodium dodecyl sulfate (SDS) as a surfactant in a two-phase extraction. 5 mg of MP was dissolved in 500 μL DCM, mixed with 250 μL of PBS + SDS using a vortex mixer, and centrifuged (5,000 g, 10 min, 4°C) to separate the phases. The aqueous phase was collected (200 μL), and the extraction process was repeated 2 more times, with 250 μL of PBS + SDS collected for the third extraction. TGF-β and IL-2 concentrations were measured using enzyme-linked immunosorbent assay (ELISA) according to manufacturer’s instructions (R&D Systems) and used to calculate drug loading (nanograms of drug per mg of microparticles). For rapamycin, drug was extracted by dissolving MP (5 mg) in acetonitrile (500 μL). Drug concentration and subsequently drug loading was calculated by measuring absorbance (278 nm) using a microplate reader (SpectraMax M5, Molecular Devices, Sunnyvale, CA) and comparing values to a standard curve of rapamycin in acetonitrile.

MP release kinetics were assessed by dissolving 10 mg of MP in 1 mL of release solution, incubating at 37°C with end-over-end rotation, and collecting samples with solution replacement at indicated time points. PBS with 1% w/v bovine serum albumin (BSA) was used as release solution for TGF-β and IL-2, and PBS with 0.02% v/v Tween-80 was used as release solution for rapamycin. TGF-β and IL-2 concentrations were assessed by ELISA and rapamycin concentration was assessed by microplate reader (absorbance 278 nm). These concentrations were then used to calculate cumulative release (ng drug/mg MP).

### Mice

Male DBA/1J mice were purchased from The Jackson Laboratory, Bar Harbor, ME), and used at 8–10 weeks of age. A single gender of mice (male) was used due to gender differences in arthritis severity in the CIA model [58,59]. All animal experiments were approved by the Institutional Animal Care and Use Committee at the University of Pittsburgh (Protocol Number: 18103788) and all methods were performed in accordance with the relevant guidelines and regulations. Animal pain and distress were assessed by checking for lethargy, weight loss (20% or more), and a scruffy coat. However, as no mice exhibited these symptoms, euthanasia was never performed prior to experimental endpoints. Mice sacrificed at experimental endpoints were euthanized using carbon dioxide followed by cervical dislocation.

### Collagen-induced arthritis (CIA) initiation, treatment, and clinical scoring

CIA was initiated as previously described [11,58]. Mice were immunized subcutaneously (s.c.) at the base of the tail on Day 0 and again on Day 21 with 100 μL of a 1:1 emulsion prepared from 4 mg/mL bovine collagen II (bCII, Chondrex, Redmond, WA) dissolved in 0.1 M acetic acid, and complete Freund’s adjuvant (CFA) consisting of incomplete Freund’s adjuvant (BD, Franklin Lakes, NJ) and 4 mg/mL of M. tuberculosis H37 RA (BD). Mice were shaved and anesthetized with isoflurane for immunizations and MP treatment to facilitate injection.

Mice were injected s.c. with 300 μL of PBS, Blank MP, or TRI MP on each flank above the hind limb on Day 0 and every 4 days through Day 12. For groups receiving MP, each injection contained 15 mg of TGF-β MP and 5 mg of IL-2 MP (or corresponding Blank MP) dissolved in PBS. Injections on Days 0 and 8 also contained 15 mg of rapamycin MP (or corresponding Blank MP). In a pilot prevention study, mice (n = 6 per group) were given daily injections (Day 0–13) on each flank above the hind limb with 100 μL of PBS, TRI Low Dose (2 ng TGF-β, 1 μg rapamycin, and 2 ng IL-2), or TRI High Dose (20 ng TGF-β, 10 μg rapamycin, and 20 ng IL-2) instead of MP.

For CIA prevention studies, mice (n = 24 per group for MP or n = 6 per group for soluble factor pilot study) were anesthetized and paws were imaged at the indicated time points between Day 26 and Day 40 so that they could be scored by a blinded individual. A clinical scoring similar to the one previously described [11,16] was used. Each paw was scored from 0–4 based on the following scale: 0 –no redness or swelling; 1 –a single digit swollen, 2 –two or more digits swollen, but no footpad/palm or ankle/wrist swelling; 3 –two or more digits swollen, and some footpad/palm or ankle/wrist swelling; 4 –all digits swollen, and severe footpad/palm and ankle/wrist swelling. The scores for each paw were summed, giving a maximum score of 16 per mouse.

### Microcomputed tomography (micro-CT) imaging and analysis

On Day 52–60, mice (n = 12 per group) selected prior to study initiation for imaging were sacrificed and hind paws were fixed in 4% formaldehyde (Thermo Fisher Scientific, Waltham, MA). The endpoint for this experiment was chosen to provide a sufficient duration of paw inflammation for bone erosion to occur [9]. Micro-CT scanning was performed using an Inveon multimodal scanner (Siemens, Washington, D.C.) at 23 μm isotropic voxel size, with 360 projections, voltage of 80 kV, and current of 500 μA. The open source program ITK-SNAP [60] (www.itksnap.org) was used to reconstruct three-dimensional images and to calculate the bone volume within an arbitrary distance of the metatarsophalangeal (MTP) joints (40 voxels or 920 μm on either side of the joint) similar to a previously described method [9]. Joint bone volume for each hind paw was calculated by summing the 5 MTP volumes. Surface meshes from the three-dimensional images made in ITK-SNAP were exported and surface area was calculated using the Meshmixer program.

### Measurement of CII antibody titer

Between Day 40–42, mice selected prior to study initiation (n = 12 per group) for serum collection were anesthetized with isoflurane and blood was collected via the retro-orbital vein. Serum was obtained by allowing blood to clot for a minimum of 30 minutes followed by centrifugation (1,000 g, 10 min) and collection of the supernatant.

ELISAs were performed as previously described [58], 96 well plates were coated overnight at 4°C with 5 μg/mL bCII in Tris-HCl (0.05 M)-NaCl (0.2 M) buffer (pH 7.4). Plates were washed with 0.05% v/v Tween-20 in PBS between all steps prior to the use of stop solution. Plates were blocked with 2% w/v BSA for 1 hr, and serum or a monoclonal anti-CII antibody used as standard (clone 2B1.5, Invitrogen, Carlsbad, CA) were serially diluted in steps of 5x from 500 fold to ~1.5 x 10^6^ fold and added in duplicate for 2 hrs. Horseradish peroxidase (HRP) conjugated goat anti-mouse-IgG (Invitrogen) at 1 μg/mL or HRP conjugated goat anti-mouse-IgG2a at 0.25 μg/mL was added for 1 hr, followed by TMB substrate (substrate reagent pack, R&D systems) for 20 min, and sulfuric acid stop solution (R&D systems). Absorbance was measured using a microplate reader (450 nm, subtracting background absorbance at 540 nm).

Antibody titer was defined as the dilution corresponding to the half-maximal absorbance in the linear section of the dilution curve [29], which was calculated as the IC50 value using a non-linear four parameter regression. Normalized titer was calculated by dividing the titer by that of the 2B1.5 antibody standard for a given plate.

### Measurement of regulatory T cell levels and phenotype in lymphoid tissue

To characterize regulatory T cells in the lymph node (LN) and spleen, mice (n = 6 per group) were immunized with bCII and injected with PBS, Blank MP, or TRI MP as described above. An endpoint of Day 15 was used for assessing draining LN (inguinal LN, iLN) T cells in order to understand the initial response immediately following MP administration, as CII-specific cells initially responding to immunization may traffic elsewhere by later timepoints. On Day 15 mice were sacrificed and the iLN and spleen were removed and ground to single cell suspensions using 70 μm filters. RBC lysis was performed on spleens with RBC lysis buffer (eBioscience, San Diego, CA), and representative samples of iLN and spleen were counted. Cells (approximately 10 million/mL) were stained with dump channel biotinylated antibodies—CD8a, CD11b, CD11c, CD19,CD45R/B220, TCR γ/δ, and F4/80 (BioLegend, San Diego, CA)—followed by BV786-Streptavidin (BD), Fc block (eBioscience), fixable viability dye (eBioscience), and for CD4 (RM4-5;BD), CD25 (PC61;BD), CD73 (TY/11.8;eBioscience), LAP (TW7-16B4; eBioscience), and CTLA-4 (UC10-4F10-1;BD). Cells were then fixed/permeabilized (FoxP3/Transcription Factor Staining Buffer Set, eBioscience), stained for FoxP3 (FJK-16s;eBioscience) and Tbet (4B10; BD), and run on a flow cytometer (Aurora, Cytek Biosciences, Barboursville, VA) and analyzed using FlowJo software (Tree Star, Ashland, OR) with gates based on isotype and single-color controls.

### Localization of inhibited T cell proliferation

To assess the effects of TRI MP on T cell proliferation and the localization of those effects, mice (n = 6 per group) were immunized with a non-arthritic antigen on one flank and immunized with collagen and TRI MP on the opposite flank. Specifically, mice were immunized s.c. on Day 0 on the left flank with 100 μL of a 1:1 emulsion prepared from 2 mg/mL Keyhole limpet hemocyanin (KLH, Sigma Aldrich) and CFA prepared as described above. Mice were also immunized on the right side on Day 0 by the base of the tail with bCII and given injections of PBS, Blank MP, or TRI MP every 4 days through Day 12 as described above. On Day 15, mice were sacrificed and the left and right iLN were removed and separately ground to single cell suspensions using 70 μm filters. Cells were stained with Fc block, fixable viability dye, and for CD4, CD25, fixed/permeabilized (FoxP3/Transcription Factor Staining Buffer Set, eBioscience), and then stained for Ki67 (SolA15;eBioscience) and Tbet (O4-06;BD). Counting beads (Thermo Fisher Scientific) were added, then samples were run on a flow cytometer (LSRII, BD) and analyzed using FlowJo (Tree Star) with gates based on isotype and single-color controls.

### Assessment of immune infiltrate in arthritic paws

Between Day 40–42, mice selected prior to study initiation for immune cell extraction from the paws were sacrificed. Paws were collected and immune cells were isolated as previously described [61]. Digits were removed and bone marrow was flushed with media, then paws (including digits) were chopped up and incubated in digestion media, cDMEM with 1 mg/mL collagenase (Sigma Aldrich) and 2.4 mg/mL hyaluronidase (Sigma Aldrich), at 37°C for 1 hr with shaking. Digested paws were then mashed and washed in 70 μm filters to create single cell suspensions. Two different staining panels were performed. In one panel (n = 12 per group), cells were stained with Fc block, fixable viability dye, and for CD45 (30-F11;eBioscience), CD4, CD25, fixed/permeabilized (FoxP3/Transcription Factor Staining Buffer Set, eBioscience), and then stained for FoxP3. In a second panel (n = 6 per group), cells were stimulated with 5 ng/mL PMA (Sigma Aldrich) and 500 ng/mL Ionomycin (Sigma Aldrich) with Golgi-Plug protein transport inhibitor (BD) for 4 hrs at 37°C. Cells were then stained with Fc block, fixable viability dye, and for CD45, CD3e (145-2C11;BD), CD19 (1D3;BD),CD11b (M1/70;eBioscience), Ly-6G (1A8-Ly6g;eBioscience), Ly-6C, (HK1.4;eBioscience), fixed/permeabilized (FoxP3/Transcription Factor Staining Buffer Set, eBioscience), and then stained for TNF-alpha (MP6-XT22;eBioscience). Counting beads (Thermo Fisher Scientific) were added, then samples were run on a flow cytometer (LSRII or Fortessa, BD) and analyzed using FlowJo (Tree Star) with gates based on isotype and single-color controls.

### Statistical analysis

Statistical analyses were performed with GraphPad Prism v7 (San Diego, CA). Data are presented as mean ± SEM and the following cutoffs were used for significance: * p < 0.05, ** p < 0.01, *** p < 0.001, **** p < 0.0001. For arthritis incidence curves, a Log-rank (Mantel-Cox) test was ran comparing all curves. Since this was significant, Long-rank (Mantel-Cox) test were performed for each individual comparison, and p values were multiplied by the number of comparisons made (3).For arthritis clinical score curves, a two-way mixed effects ANOVA (for time as a repeated measure and treatment group) was performed, followed by Tukey post-hoc analysis to compare the mean of every group with the mean of every other group at each time point. The ROUT outlier test with the most stringent threshold for outlier removal (Q = 0.1%) was used to remove outliers from the graph of normalized antibody titers. For all plots assessing a correlation with arthritis scores, the Spearman r correlation coefficient was calculated and a two-tailed p value was used to determine the significance of the correlation. All other graphs had 3 treatment groups and were analyzed by one-way ANOVA, followed by Tukey post-hoc analysis in order to compare the mean of every group with the mean of every other group.

## Results

### TRI MP treatment prevents induction of arthritis

**Fig 1:**
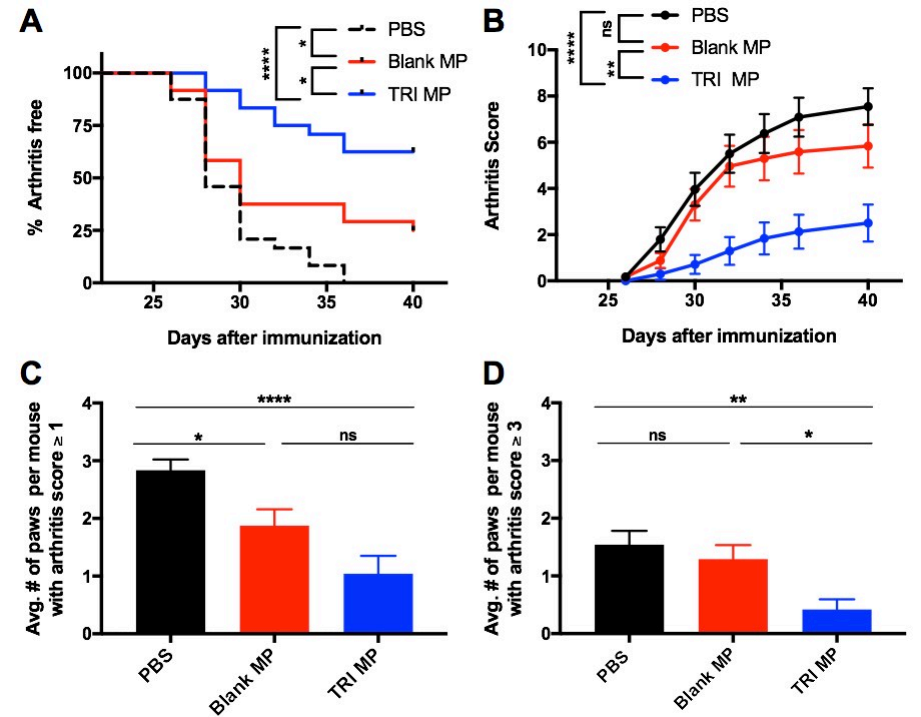
TRI MP administration reduces incidence and severity of CIA onset. **A)** Survival curve indicating percentage of mice that remained arthritis free (score of 0 for all paws). **B)** Arthritis scores over time. A two-way ANOVA was performed, followed by Tukey post-hoc analysis to compare the mean of every group with the mean of every other group at each time point. Significance labels apply to all time points from Day 32 on. **C-D)** Average number of paws per mouse with arthritis score greater than or equal to specified threshold of 1 (C) or 3 (D) at Day 40. n = 24 mice per group, data presented as mean ± SEM, and the following cutoffs were used for significance: * p < 0.05, ** p < 0.01, **** p < 0.0001.

TRI MP morphology, size, and drug release kinetics (S1 Fig) were similar to those previously reported [54]. The dose of MP administered for CIA prevention was chosen based on MP release (S1 Fig) in order to approximate the effect observed in a pilot CIA prevention study using daily local injection of un-encapsulated TRI (S2 Fig). In the MP CIA prevention study, PBS treated mice had less than 50% of mice remaining arthritis free by Day 28 and all mice had developed arthritis by Day 36 (Fig 1A). Blank MP, or vehicle control, treated mice had less than 50% of mice remaining arthritis free by Day 30, and 25% of mice remining arthritis free at the study endpoint (Fig 1A). In comparison to these groups, TRI MP had a significantly improved survival curve (Mantel-Cox, p < 0.0001 and p < 0.05 respectively), with 62.5% of mice remaining arthritis free at the study endpoint (Fig 1A). When the clinical arthritis score was assessed, TRI MP significantly prevented the development of disease relative to both PBS (Two-way ANOVA, Tukey post-hoc, p < 0.0001) and Blank MP (Two-way ANOVA, Tukey post-hoc for treatment group, p < 0.01) treatment at all timepoints past Day 32 (Fig 1B). These differences were 2-3x in magnitude with TRI MP treatment resulting in an average arthritis score of 2.5 at Day 40, while PBS and Blank MP treatment led to average arthritis scores of 7.5 and 5.8 respectively (Fig 1B). To demonstrate how TRI MP treatment influenced the number and severity of inflamed paws, results were also presented in terms of number of affected paws per mouse. Relative to PBS treatment, TRI MP treatment significantly reduced the number of paws per mouse with arthritis (Fig 1C), as well as the number of paws per mouse with severe arthritis (Fig 1D). Where severe arthritis (arthritis score ≥ 3 per paw) was defined by the involvement of footpad/ankle swelling. Taken together, these data show that TRI MP was able to significantly inhibit the incidence and severity of arthritis in a prevention model.

### Correlation demonstrated between arthritis clinical score and bone erosion

**Fig 2:**
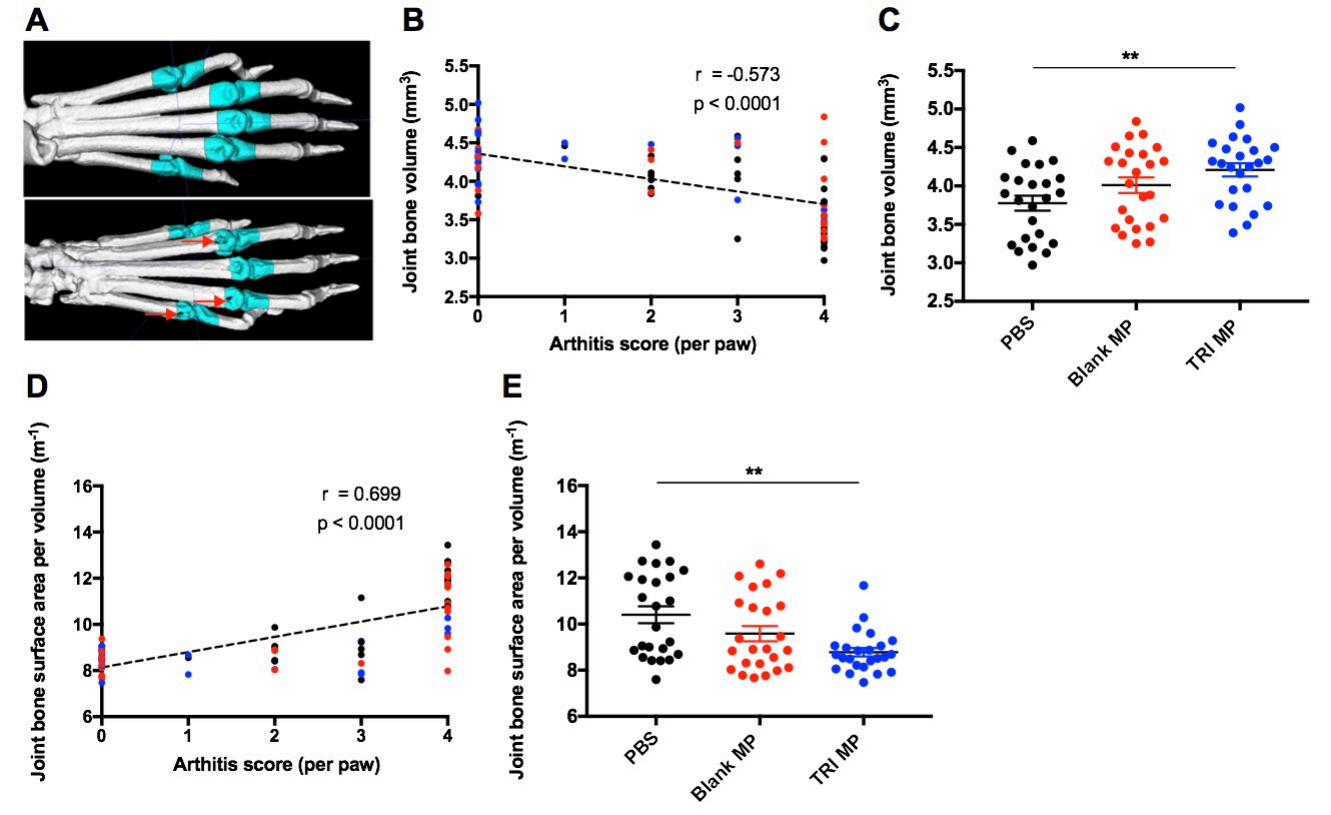
Lower arthritis score correlates with less bone erosion. **A)** Representative 3D reconstructions of micro-CT scans showing a paw without bone erosion (top) and a paw with severe bone erosion (bottom). Joint masks indicated in cyan were the regions used to calculate joint bone volume. **B)** Joint bone volume versus arthritis score at Day 40 for individual hind paws. Color coded based on treatment group: black–PBS, red–Blank MP, blue–TRI MP. Spearman correlation coefficient and p value for correlation are indicated. **C)** Average joint bone volume by treatment group. **D)** Joint bone surface area to volume ratio versus arthritis score at Day 40 for individual hind paws. Color coded based on treatment group: black–PBS, red–Blank MP, blue–TRI MP. Spearman correlation coefficient and p value for correlation are indicated. **E)** Average joint bone surface area to volume ratio by treatment group. n = 24 paws (12 mice) per group, data presented as mean ± SEM, and the following cutoffs were used for significance: ** p < 0.01.

To determine if the reduction in paw inflammation observed with TRI MP administration was associated with less bone erosion, micro-computed tomography (CT) scans were performed on fixed hind paws from mice sacrificed between Day 52 and Day 60. An experimental timeline illustrating the different cohorts for mice (used for different experimental endpoints) can be found in S3 Fig. Visible full-thickness bone erosions could be detected at the MTP joints in some paws with high clinical arthritis scores (Fig 2A). Quantification of the relationship between MTP joint bone volume and arthritis score for an individual paw demonstrated a negative, moderate strength (Spearman r = -0.573), and significant (p < 0.0001) correlation (Fig 2B). Notably there is some variability in the joint bone volume among paws that had arthritis scores of zero at Day 40. While some of this may be natural variation present in healthy paws (i.e. bone volumes of ~ 4mm^3^ – 5mm^3^), some of the lower bone volume measurements in this group may reflect the delayed emergence of arthritis between Day 40 and Day 60 in corresponding mice. Despite this variability, the moderate strength and significant correlation observed suggest that on average the arthritis score is still a good predictor of bone erosion. When the data is presented by treatment group, the TRI MP group has significantly (one-way ANOVA, Tukey post-hoc, p < 0.001) more joint bone volume than the PBS group (Fig 2C). Depending on the severity, an arthritic bone erosion should theoretically result in a loss of joint bone volume (V) and/or an increase in joint bone surface area (SA) due to the irregular nature of bone erosions. Together this would result in an increased surface area to volume ratio (SA/V). As expected, there was a positive, moderate strength (Spearman r = 0.699), and significant (p < 0.0001) correlation between MTP joint bone surface area to volume ratio and arthritis score (Fig 2D). Likewise relative to PBS treatment, TRI MP treatment significantly (one-way ANOVA, Tukey post-hoc, p < 0.001) prevented the increased joint bone surface area to volume ratio associated with arthritis (Fig 2E). Together these findings demonstrate that a reduced arthritis score was associated with protection from bone erosion, and on average TRI MP treated mice exhibited less bone erosion.

### Auto-antibodies are reduced in mice that are administered TRI MP

**Fig 3:**
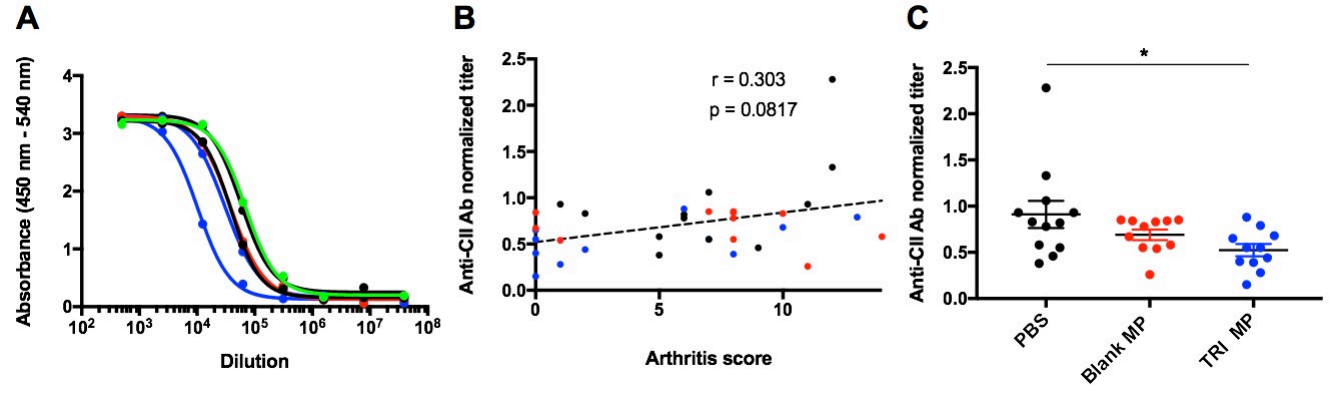
TRI MP administration lowers level of anti-collagen II IgG antibodies. **A)** Representative serial dilution curves with each curve corresponding to a single mouse, black–PBS treated mouse, red–Blank MP treated mouse, blue–TRI MP treated mouse, green–monoclonal anti-collagen II (CII) antibody (Ab) (Clone 2B1.5) used as standard. **B)** Normalized anti-CII IgG Ab titer versus arthritis score. Ab titer was defined as the dilution corresponding to the half-maximal absorbance in the linear section of the dilution curve, or the IC50 value using a non-linear four parameter regression. Normalized titer was calculated by dividing the titer by that of the 2B1.5 Ab standard for a given plate. Color coded based on treatment group: black–PBS, red–Blank MP, blue–TRI MP. Spearman correlation coefficient and p value for correlation are indicated. C) Average normalized anti-CII IgG Ab titer by treatment group. n = 12 mice per group, data presented as mean ± SEM, and the following cutoffs were used for significance: * p < 0.05.

To begin to understand the mechanism by which TRI MP is acting, serum taken on Day 40 was used in indirect ELISAs with bCII as the antigen to measure levels of anti-CII IgG antibodies (Ab). Representative serial dilution curves (Fig 3A) show one TRI MP mouse (blue) with a particularly left-shifted curve, and thus reduced anti-CII IgG Ab titer. Ab titer was normalized to the titer of a monoclonal CII Ab included on each plate to account for plate-to-plate variability. A plot of normalized anti-CII IgG Ab titer vs. arthritis score had a weak (Spearman r = 0.303) and non-significant (p = 0.0817) correlation (Fig 3B). However, TRI MP treatment significantly (one-way ANOVA, Tukey post-hoc, p < 0.05) lowered the average anti-CII IgG Ab titer by approximately 40% relative to PBS treatment (Fig 3C). These results demonstrate that TRI MP significantly reduced the level of an arthritis causing auto-antibody but did not completely block auto-antibody generation even in mice that had no signs of arthritis. The lack of correlation between anti-CII IgG Ab titer and arthritis clinical score suggests that the mechanism of TRI MP action is not a reduction of the concentration or affinity of total anti-CII IgG Ab. While the anti-CII IgG level has been associated with CIA disease severity in a few studies [29,39], there is also evidence that the percentage of anti-CII IgG that is of the Th1 associated IgG2a isotype [62] and not the overall IgG level predicts susceptibility to CIA since IgG2a is associated with complement system activity [63,64]. Therefore, anti-CII IgG2a Ab titers were also assessed. There was no correlation between anti-CII IgG2a Ab titers and arthritis score and no differences in anti-CII IgG2a Ab titers between treatment groups (S4 Fig).

### TRI MP treatment increases a CD4^+^ T cell population with elevated regulatory markers in the draining lymph node and spleen

**Fig 4:**
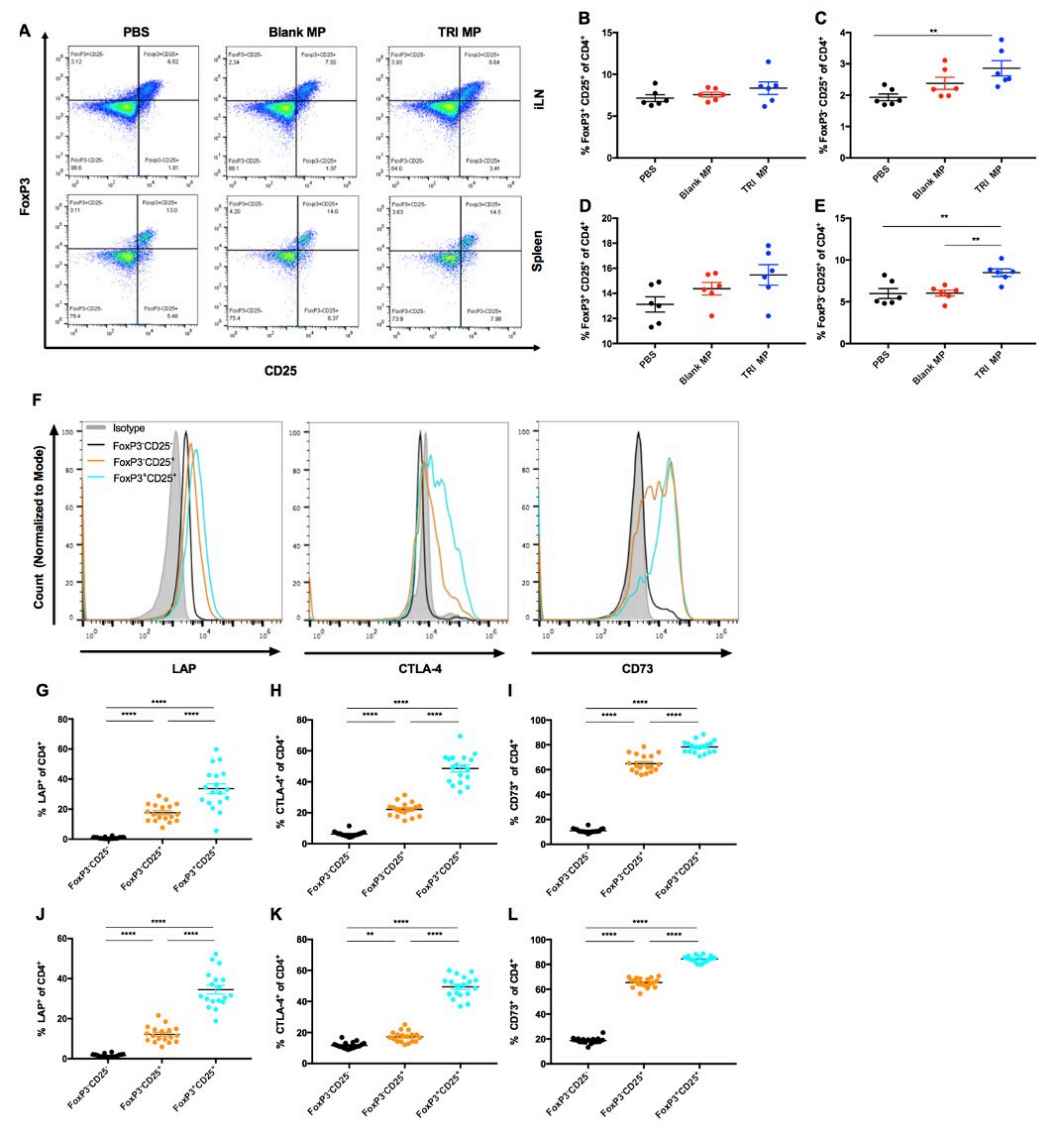
TRI MP leads to more CD25^+^FoxP3^-^ T cells, which express elevated levels of LAP, CTLA-4, and CD73. **A)** Representative pseudocolor plots of CD25 expression versus FoxP3 expression for CD4^+^ cells from the iLN (top row) or spleen (bottom row) of mice treated with PBS (left column), Blank MP (center column), or TRI MP (right column). **B-E)** Quantification of plots from A, showing percentage of CD4^+^ cells that are FoxP3^+^CD25^+^ **(B, E)** or FoxP3^-^CD25^+^ **(C, D)** by treatment group for the iLN **(B, C)** and spleen **(D, E)**. **F)** Representative histogram plots of LAP (left), CTLA-4 (middle), and CD73 (right) expression for the isotype control (shaded gray), the FoxP3^-^CD25^-^ population (black), the FoxP3^-^CD25^+^ population (orange), and the FoxP3^+^CD25^+^ (cyan) population (from a PBS treated mouse). **G-L)** Quantification of the percentage of CD4^+^ T cell populations that are LAP^+^ **(G,J)**, CTLA-4^+^ **(H, K)**, or CD73^+^ **(I, L)** relative to isotype control. Presented by CD4^+^ T cell population (FoxP3^-^CD25^-^, FoxP3^-^CD25^+^, and FoxP3^+^CD25^+^) for the iLN **(G-I)** and spleen **(J-L)**. n = 6 mice per treatment group and n = 18 mice per CD4^+^ T cell population group, data presented as mean ± SEM, and the following cutoffs were used for significance: ** p < 0.01, *** p < 0.001, **** p < 0.0001.

To investigate whether regulatory T cells could be playing a role in TRI MP prevention of CIA, mice were immunized with bCII, injected with PBS, Blank MP, or TRI MP every 4 days, and sacrificed on Day 15. While there was not a significant increase in the levels of FoxP3^+^CD25^+^ Tregs in the draining lymph node (inguinal, iLN) (Fig 4A and 4B) or spleen (Fig 4A and 4D) of TRI MP treated mice relative to controls, there was a significant increase (one-way ANOVA, Tukey post-hoc, p < 0.01) in FoxP3^-^ CD25^+^ T cells relative to PBS treated mice in the iLN (Fig 4C) and spleen (Fig 4E). Likewise, no significant increase in FoxP3^+^ Tregs was observed at a later time point (Day 35) in the pilot study with daily injections of un-encapsulated TRI factors (S2 Fig). Several markers associated with regulatory T cell function were also assessed to evaluate how their expression on the FoxP3^-^ CD25^+^ population compared to that of conventional CD4^+^ T cells (FoxP3^-^ CD25^-^) and Tregs (FoxP3^+^ CD25^+^), as well as whether TRI MP led to evaluated expression of these markers relative to control treatments on either the FoxP3^-^ CD25^+^ or Treg populations. The analyzed markers included: latency-associated peptide (LAP), part of the latent TGF-beta complex; CTLA-4, a checkpoint molecule that blocks CD80/86 co-stimulation; and CD73, an enzyme which degrades AMP to immunosuppressive adenosine. When mice from all treatment groups were pooled together in the analysis, the FoxP3^-^ CD25^+^ population had significantly (One-way ANOVA, Tukey post-hoc for T cell population, p < 0.01 or p <0.0001) higher expression of LAP, CTLA-4, and CD73 than the conventional CD4^+^ T cells (FoxP3^-^ CD25^-^) population in both the iLN and spleen (Fig 4F–4L). However, while TRI MP treatment resulted in significantly elevated expression of CD73 for the iLN FoxP3^-^ CD25^+^ population, TRI MP led to trends toward reduction (and one significant example) of LAP and CTLA-4 expression for the FoxP3^-^ CD25^+^ and/or Treg (FoxP3^+^ CD25^+^) populations in the iLN and spleen (S5 Fig). It is possible that reduced inflammation in TRI MP treated mice prevented the upregulation of these suppressive markers. Tbet expression was also assessed, but an appreciable Tbet^+^ population was not detected (S6 Fig). While TRI MP treatment did not increase the levels of conventional FoxP3^+^ Tregs or increase expression of suppressive markers on these cells, it did increase a population of activated CD4^+^ T cells (FoxP3^-^CD25^+^) that had elevated levels of suppressive markers.

### The effects of the dose of TRI MP administered are not localized to the draining lymph node

**Fig 5:**
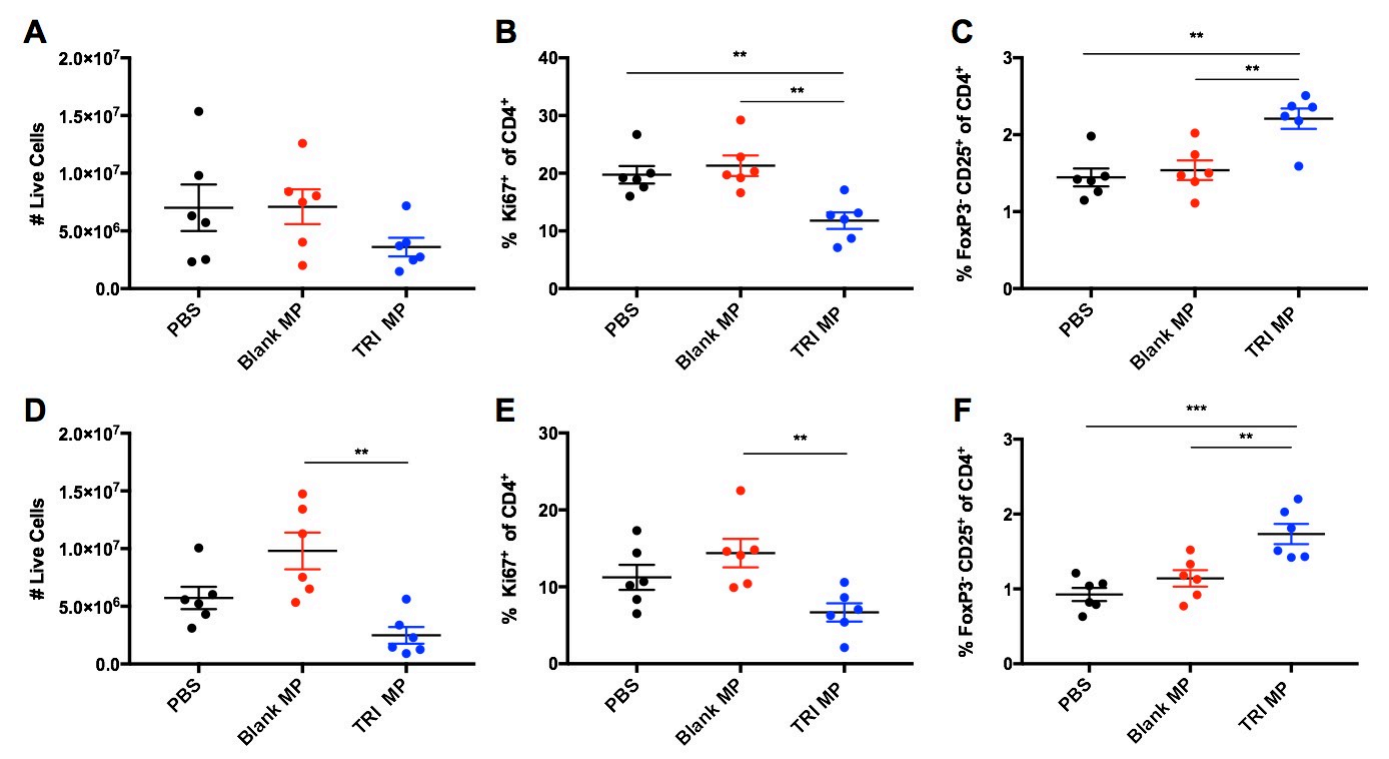
TRI MP reduces conventional T cell expansion and expands regulatory population not only for the draining lymph node, but also for a non-arthritic immunization in the contralateral lymph node. **A)** Number of live cells in draining iLN as determined using counting beads. **B)** Percentage of CD4^+^ T cells expressing the proliferation marker Ki67 in the the draining LN. **C)** Percentage of CD4^+^ T cells that are FoxP3^-^CD25^+^ in the draining LN. **D-F)** Same as A-C, but for the contralateral LN instead of the draining LN. n = 6 mice per group, data presented as mean ± SEM, and the following cutoffs were used for significance: ** p < 0.01, *** p < 0.001.

In order to evaluate the role of TRI MP suppression of T cell proliferation in arthritis protection as well as the localization of this immunosuppression, mice were immunized with KLH on one flank and immunized with bCII along with PBS, Blank MP, or TRI MP on the other flank. The iLN of the TRI MP treated flank had a trend towards reduced cell numbers, and significantly (One-way ANOVA, Tukey post-hoc, p < 0.01) reduced proliferation of CD4^+^ T cells (Fig 5A and 5B). There also was an increase in the FoxP3^-^CD25^+^ population (Fig 5C) consistent with Fig 4C. However, the contralateral limb in TRI MP treated mice also had the response to immunization suppressed to a similar degree. The contralateral iLN of the TRI MP group had a trend towards reduced cell numbers and reduced proliferation, with significant differences (One-way ANOVA, Tukey post-hoc, p < 0.01) observed relative to the Blank MP group (Fig 5D and 5E). There was also a significant increase (One-way ANOVA, Tukey post-hoc, p < 0.01) in the FoxP3^-^CD25^+^ population in the contralateral iLN (Fig 5F). These results suggest that the actions of TRI MP were not localized to the draining LN, as similar levels of reduced cellular proliferation and an increased regulatory population were observed in both the draining and contralateral iLN.

### Lower arthritis score associated with less immune infiltrate and inflammatory cytokine in the paws

**Fig 6:**
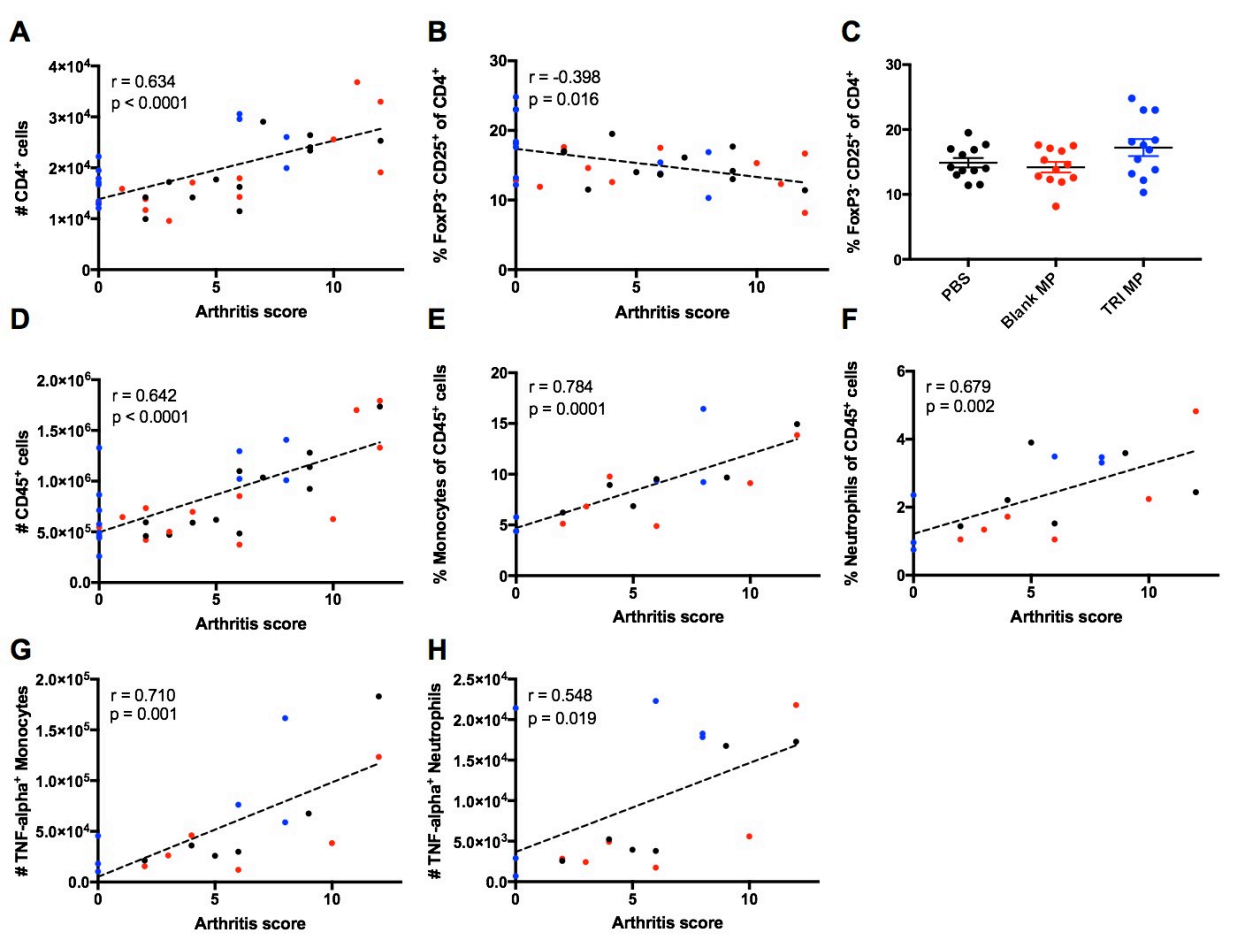
Lower arthritis score correlates with less CD4+ T cells, a higher proportion of regulatory cells, and less inflammatory innate immune cells in the paws. **A,B,D-H)** Indicated parameter of the paw immune infiltrate versus arthritis score (Day 40–42). Spearman correlation coefficient and p value for correlation are indicated. n = 6–12 mice per group. These include the number of CD4^+^ T cells (**A**), the percentage of CD4^+^ T cells that are FoxP3^-^ and CD25^+^ (**B**), the number of CD45^+^ immune cells (**D**), the percentage of CD45^+^ cells that are monocytes/macrophages (CD11b^+^Ly-6G^-^Ly-6C^+^) (**E**), the percentage of CD45^+^ cells that are neutrophils (CD11b^+^Ly-6G^+^) (**F**), the number of TNF-α expressing monocytes/macrophages (**G**), and the number of TNF-α expressing neutrophils (**H**). **C)** Percentage of CD4^+^ T cells that are FoxP3^-^CD25^+^ by treatment group. n = 12 mice per group, data presented as mean ± SEM.

To assess how TRI MP treatment altered the amount and characteristics of immune infiltrate in the inflamed paws themselves, immune cells were extracted from paws between Day 40–42. Since TRI MP was shown to reduce CD4^+^ T cell proliferation and expand a FoxP3^-^CD25^+^ population expressing suppressive markers in the draining LN (Figs 4 and 5), the accumulation of CD4^+^ T cells in the paws and fraction of them that were FoxP3^-^CD25^+^ was assessed. A moderate (Spearman r = 0.634) and significant (p < 0.0001) positive correlation was observed between the number of CD4^+^ T cells in the paws and the arthritis score (Fig 6A). While mice with lower arthritis scores had fewer number of CD4^+^ T cells in the paws, a larger percentage of these CD4^+^ T cells were FoxP3^-^CD25^+^ (Fig 6B). Although TRI MP treated mice did not have a significantly different FoxP3^-^CD25^+^ cell population relative to PBS and Blank MP controls (Fig 6C), the average for TRI MP was slightly larger driven by three TRI MP treated mice with arthritis scores of zero and greater than 20% of CD4 T cells expressing the FoxP3^-^CD25^+^ phenotype (Fig 6C). Notably, the percentage of CD4^+^ T cells expressing FoxP3 was not significantly correlated with arthritis score or significantly increased with TRI MP treatment (S7 Fig). Given the paradigm of auto-antibodies and CD4^+^ T cells promoting myeloid cell recruitment and expansion in CIA, the size of the overall immune infiltrate in the paws and levels of monocytes/macrophages and neutrophils were assessed. There was a significant (p < 0.0001) positive correlation between the amount of immune infiltrate in the paws, as defined by CD45 expression, and the arthritis score (Fig 6D). Not only did the number of immune cells present in the paws increase with higher arthritis scores, but the composition of the CD45^+^ immune population changed as well. The percentages of monocytes/macrophages (CD11b^+^Ly-6G^-^Ly-6C^+^) [65] and neutrophils (CD11b^+^Ly-6G^+^) among CD45^+^ cells were significantly (p = 0.0001 and p = 0.002 respectively) and positively correlated with arthritis score (Fig 6E and 6F). The percentage of monocytes/macrophages and neutrophils in the paws of bCII-immunized mice was also noticeably higher than that of un-immunized mice (S7 Fig). Due to the role of TNF-α in causing paw redness and swelling, myeloid cell expression of TNF-α was also measured. Both the number of TNF-α^+^ monocytes/macrophages and the number of TNF-α^+^ neutrophils were significantly (p = 0.001 and p = 0.019) and positively correlated with arthritis score (Fig 6G and 6H). Taken together, these findings are consistent with a scenario in which mice with low arthritis scores have less CD4^+^ T cell infiltrate and/or a higher proportion of regulatory FoxP3^-^CD25^+^ cells, resulting in reduced infiltrate of myeloid cells and less production of an inflammatory cytokine responsible for paw redness and swelling.

## Discussion

New therapeutic approaches to RA are necessary as a large number of patients do not respond sufficiently to existing treatments. In particular, approaches aiming to maintain or restore Treg–Teff balance are of considerable interest because of the role this balance has in influencing disease progression, both in RA and the murine model of CIA. We have previously explored microparticle formulations that expand Tregs and limit Teff levels, resulting in disease prevention or therapeutic treatment in several preclinical models. Here we evaluated the ability of TRI MP to prevent the development of arthritis in the CIA model, and explored the mechanism behind disease prevention. Mice given s.c. TRI MP injections every four days between Day 0–12 following bCII immunization had significantly reduced incidence of arthritis and severity of arthritis relative to both PBS and Blank MP treated control groups (Fig 1). The protection provided by TRI MP not only served to block tissue swelling, but also prevented bone erosion in the digits relative to PBS but not relative to Blank MP (Fig 2). While this may in part reflect some protective effect of Blank MP as discussed below, the lack of significant difference between Blank MP and TRI MP in bone erosion also likely reflects some limitations of the bone erosion assessment given the robust differences observed between Blank MP and TRI MP in Fig 1. First although all PBS treated mice developed arthritis, only a small fraction had paws with substantial bone erosions. While this is likely a natural reflection of the fact that sufficient severity and duration of inflammation must occur to result in bone erosion, it means that differences between treatment groups will be accordingly harder to detect. Secondly, there was a relatively large degree of variability in the bone volume measurements of mice without arthritis relative to the magnitude of bone volume reduction in mice with bone erosions (Fig 2B). The assessment of bone surface area to volume ratio as opposed to bone volume resulted in a stronger correlation with arthritis score and a larger trend towards a difference between Blank MP and TRI MP (Fig 2D and 2E). This may have been due an ability of the surface area to volume ratio to partially mitigate these limitations. For example, minor bone erosions would be expected to be disproportionally detected by surface area and the surface area to volume ratio may help reduce natural variability in the size of healthy (arthritis score of zero) joints. Taken together these factors of a high bar for detection and high variability may have contributed to the lack of significant difference observed for bone erosions between Blank MP and TRI MP based on the sample size studied. However, a substantial and significant difference was still demonstrated with TRI MP treatment compared to both PBS and Blank MP for the primary endpoints of the CIA model, arthritis incidence and arthritis score (Fig 1A and 1B).

The first step in examining the mechanism by which TRI MP achieved these preventative effects was assessing levels of anti-CII auto-antibodies. While TRI MP significantly lowered titers of anti-CII IgG Ab relative to the PBS control, there were still similar titers in mice who were arthritis free and those who developed severe arthritis (Fig 3). Likewise, no correlation between titers of anti-CII IgG2a and clinical arthritis score was observed (S4 Fig). This suggests that TRI MP was either affecting other aspects of the Ab response, such as epitope spreading, and/or affecting immune cell recruitment/expansion in the paws. The impact of TRI MP administration on the CD4^+^ T cell population was assessed next since TRI MP has been proven to influence CD4^+^ Treg and Teff levels in other disease models [53–55], and CD4^+^ T cells contribute to CIA disease severity independently of helping with Ab production [12]. While TRI MP treatment did not result in increased levels of canonical FoxP3^+^ Tregs in the draining LN or spleen at the Day 15 time point, it did result in increased levels of a FoxP3^-^CD25^+^ T cell population with elevated expression of several suppressive molecules utilized by Tregs including LAP, CTLA-4, and CD73 (Fig 4). TRI MP also had an anti-proliferative effect, reducing immunization-induced expansion of LN cell numbers and the proliferation of CD4^+^ T cells in the LN at Day 15 (Fig 5). To understand how these early TRI MP induced changes in the periphery protected against the development of arthritis between Days 26–40, paw immune infiltrate was analyzed at the experimental endpoint. Mice with lower arthritis scores had less CD4^+^ T cells in the paws, but a larger percentage of cells were FoxP3^-^CD25^+^ (Fig 6). Additional correlations showed that lower arthritis scores were associated with reduced overall immune infiltration, reduced myeloid cell representation among the infiltrate, and less TNF-α producing myeloid cells (Fig 6). These findings are consistent with a mechanism in which TRI MP decreases arthritis score by limiting immunization-induced expansion of CD4^+^ T cells directly and/or through an increased regulatory FoxP3^-^CD25^+^ population in the periphery, resulting in less CD4^+^ T cell recruitment and/or the migration of FoxP3^-^CD25^+^ T cells to the paws, and in turn less recruitment and activation of myeloid cells to produce arthritis causing inflammatory cytokines. Although significant correlations in agreement with this mechanism were observed for Fig 6, when the data was presented by treatment group significant differences were not observed between TRI MP and other treatment groups (Fig 6 and S7 Fig). This may be because of a lower sample size in this experiment than the prevention study, less separation between the TRI MP and control group arthritis scores in the cohort used for this experiment, and/or because the phases of paw inflammation are dynamic with the timing of disease onset varied among mice. While significant correlations with arthritis score cannot definitely prove the order or causality of the proposed mechanism of action for TRI MP, literature on the immunological processes of CIA development supports this sequence of events [10]. While it is possible that TRI MP administration does not directly cause all of the arthritis score associated changes in paw immune infiltrate observed, if TNF-α is directly responsible for the redness and swelling measured in arthritis scores [10,18], then at the very least TRI MP must reduce TNF-α production in order to lower arthritis scores.

Previous studies investigating TRI MP have demonstrated that the combination of all three types of MP in TRI MP was more effective than any single MP factor or any dual combination of factors [53–55], so only Blank MP and PBS alone controls were evaluated here. The Blank MP control exhibits a trend in the same direction as TRI MP in several figures, including figures where Blank MP is significantly different from PBS (Fig 1A and 1C) or Blank MP is not significantly different from TRI MP (Figs 2C, 3C, and 4C). These findings may be due to the immunomodulatory properties of PLGA microparticles themselves. Notably, lactic acid from PLGA MP degradation was previously shown to inhibit dendritic cell maturation, possibly by interfering with NF-κB activation [66]. Furthermore, i.v. injected PLGA NP prevented auto-immunity by causing monocytes/neutrophils that phagocytosed them to traffic to the liver and spleen instead of the site of inflammation [67]. While the average diameter of TRI MP was approximately 15–20 μm (S1 Fig), a size likely too large to be phagocytosed by APCs, there is a relatively broad distribution of MP size with some small enough to be phagocytosed (albeit with those smaller microparticles in the distribution representing a much smaller quantity of overall % encapsulated active ingredients). These effects could be more pronounced in this model relative to past models TRI MP have been used in due to a higher dose and frequency of microparticle administration. Despite any protective effects observed with the Blank MP group, the drugs delivered by TRI MP still have a substantial and significant role in reducing arthritis incidence and severity (Fig 1A and 1B).

While TRI MP was hypothesized to increase levels of FoxP3^+^ Tregs in the CIA model based on experience with TRI MP in most other disease models, a previous TRI MP study also observed increases in a population of FoxP3^-^CD25^+^ cells similar to the one observed here and the regulatory function of this regulatory population was demonstrated through a T cell suppression assay. Specifically, in an OVA protein-specific contact hypersensitivity model, TRI MP administration led to a significant increase in the percentage of OVA-specific CD4^+^ T cells that were FoxP3^-^CD25^+^ Tbet^-^ but not a significant increase in the percentage of CD4^+^ T cells that were FoxP3^+^ [53]. Because this was an adoptive transfer model involving use of congenic (CD45.2) OT-II T cell clone, the percentage of transferred CD4^+^ T cells expressing FoxP3^+^ was negligible and over 90% of the (CD45.2^+^CD4^+^) CD25^+^ population in the draining LN of TRI MP treated mice was made up of FoxP3^-^CD25^+^ Tbet^-^ cells as opposed to FoxP3^+^CD25^+^ cells [53]. Thus, when CD45.2^+^CD4^+^ CD25^+^ T cells were sorted and shown to inhibit conventional (CD4^+^ CD25^-^) T cell proliferation in a suppression assay at ratios as low as 1 CD25^+^ T cell: 8 conventional T cells [53], it was clear that the FoxP3^-^CD25^+^ Tbet^-^ population had suppressive function. Here we observed increases in a similar population with likely regulatory function that was characterized as FoxP3^-^CD25^+^ and had elevated expression of LAP, CTLA-4, and CD73 (Fig 4). However, because of the much higher level of FoxP3^+^CD25^+^ Tregs in the CIA model, the FoxP3^-^CD25^+^ population accounts for only ~25% of the CD25^+^ population in the draining LN of TRI MP treated mice (Fig 4). Therefore, a suppression assay using CD25^+^ regulatory cells in the CIA model would be unlikely to be informative due to an inability to distinguish the suppressive contribution of the FoxP3^-^ in the presence of a much larger population of suppressive FoxP3^+^ cells. Although it cannot definitively be claimed that the FoxP3^-^CD25^+^ population observed here is not an activated effector population, the increased levels of suppressive markers expressed by this population, similarity to a verified suppressive population observed using TRI MP in a different disease model, and the observations that TRI MP provided strong CIA protection while inhibiting CD4^+^ T cell proliferation together provide strong evidence for the regulatory nature of the FoxP3^-^CD25^+^ population increased by TRI MP. The reason that an increase in FoxP3^+^ expression was not observed in this model is unclear, but may have to do with the MP dose used, MP injection location, use of CFA as the priming agent, and/or the single initiating antigen in this model as opposed to previous models eliciting more polyclonal responses.

Further developing TRI MP towards clinical use for arthritis will require dose optimization to minimize any non-specific immunosuppression. The use of subcutaneous MP delivery, depending on the drug delivered and it’s dose, may be able to keep delivery relatively localized to the injection site [68]. This is of particular interest when delivering immunomodulatory or immunosuppressive agents, so that the ability of the immune system to fight pathogens in other tissues is not impaired. A previous study evaluating hind limb allotransplantation found that TRI MP injected in the contralateral limb was not effective in prolonging graft survival relative to TRI MP injected in the transplanted limb, indicating that the immunomodulatory effects of TRI MP were restricted to the local area/antigens [55]. Here, we found that TRI MP administered on one limb reduced proliferation and expanded a regulatory population in the contralateral limb (Fig 5). The reason for this discrepancy may be the larger dose of TRI MP used in this study, and in particular, the dose of rapamycin. Unlike active TGF-β and IL-2, which have serum half-lives of only 2–4 minutes when i.v. injected [69,70], rapamycin has a serum half-life of 6 hours when i.v. injected [71] which may permit greater systemic distribution than the other TRI MP components. Although TRI MP dosing used in this study was based on a pilot using daily injections of un-encapsulated drugs and a lower TRI dose provided limited arthritis protection (S2 Fig), it is possible that further optimization of dose and delivery kinetics to use a rapamycin dose in between that of high and low tested doses and/or lowering the rapamycin dose while increasing doses of TGF-β and IL-2 yields a formulation capable of preventing CIA development without causing systemic immunosuppression. When TRI MP was previously shown to be more effective than a comparable dose of un-encapsulated TRI factors in a different model, both were given at the same frequency (one administration for a shorter timeline) [53] While the pilot experiment using the higher dose of un-encapsulated TRI factors for TRI MP dose estimation led to an arthritis score of similar magnitude to TRI MP, the sample size of this group was substantially smaller (n = 6 vs. n = 24) and the daily delivery of un-encapsulated TRI factors partially mimicked the sustained delivery role of microparticles which were given less frequently in the later prevention study. A direct comparison of TRI MP to un-encapsulated TRI factors administered with the same frequency should be evaluated in the CIA model after future optimization of TRI factor dose. Pharmacokinetic studies will ultimately be necessary to support TRI MP translation for arthritis or other indications, however radiolabeled agents may be required given the extremely small amount of cytokines released.

In summary, this study found that TRI MP was able to significantly reduce the incidence, severity, and associated bone erosion of arthritis induced by collagen II immunization. The mechanism of this protective effect involved reduced CD4^+^ T cell proliferation and an increased regulatory population in the periphery following TRI MP administration, and these changes were also reflected in the paws during arthritis onset and associated with reduced recruitment/expansion of TNF-α producing myeloid cells. The next steps in the development of TRI MP as a therapy for arthritis include identifying optimal dosing to prevent CIA without causing systemic immunosuppression and evaluating the ability of TRI MP to reverse established arthritis for clinical relevance.

## Supporting information

S1 Fig Microparticle characterization. **A-C)** Drug loading (ng/mg) for TGF-β microparticles (MP) **(A)**, IL-2 MP **(B)**, and rapamycin MP **(C)** respectively. n = 6–12 batches of MP per group, data presented as mean ± SEM**. D-F)** *In vitro r*elease kinetics for TGF-β MP **(D)**, IL-2 MP **(E)**, and rapamycin MP **(F)** respectively. Representative batch of MP shown with release samples performed in triplicate and presented as mean ± SEM. **G)** SEM images showing surface morphology of TGF-β MP, IL-2 MP, and rapamycin MP with 10 μm scale bar shown for reference. **H)** Average MP diameter measured by Coulter Counter, presented as mean ± SEM.
(TIF)

S2 Fig Pilot study using unencapsulated TRI factors. **A)** Arthritis scores over time for mice given daily injections (Day 0–13) on each flank above the hind limb with 100 μL of PBS, TRI Low Dose (2 ng TGF-β, 1 μg rapamycin, and 2 ng IL-2), or TRI High Dose (20 ng TGF-β, 10 μg rapamycin, and 20 ng IL-2. **B)** Quantification of the percentage of CD4^+^ T cells that are FoxP3^+^ in the draining (inguinal) lymph node on Day 35. n = 6 mice per group, data presented as mean ± SEM.
(TIF)

S3 Fig Experimental timelines for microparticle treated animal studies. **A)** Timeline for CIA prevention and associated endpoints. Mice (n = 24 per group) were immunized with an emulsion of complete Freund’s adjuvant (CFA) and bovine collagen II (bCII) at the base of the tail on Day 0 and subcutaneously (s.c.) injected with PBS or microparticles (MP) by both hind limbs every 4 days between Day 0 and Day 12. Mice were scored by a blinded individual for signs of arthritis between Day 26 and Day 40 (Fig 1), at which point half of mice were sacrificed and used to measure serum auto-antibodies (Fig 3) and to extract immune cells from the paws (Fig 6). The other half of mice were left until Day 52–60 to allow sufficient time for inflammation to result in bone erosion, and then sacrificed and used for micro-computed tomography (CT) (Fig 2). **B)** Timeline for measurement of regulatory T cell levels and phenotype in lymphoid tissue. Mice (n = 6 per group) treated as in A), but sacrificed at Day 15 to assess T cells at a time point close to MP administration to assess regulatory T cell levels and phenotype in the draining inguinal lymph nodes (iLN) and spleen (Fig 4). **C)** Timeline for localization of inhibited T cell proliferation. Mice (n = 6 per group) were immunized with bCII by the base of the tail on the right side only, and on the left flank an emulsion of CFA and Keyhole limpet hemocyanin (KLH) was given. Mice were treated with PBS or MP as described above, but only on the right flank. T cell responses were assessed for both the draining iLN (right side) and contralateral iLN (left side) relative to MP localization (Fig 5).
(TIF)

S4 Fig Assessment of anti-collagen II IgG 2a antibodies. **A)** Normalized anti-CII IgG2a Ab titer versus arthritis score. Ab titer was defined as the dilution corresponding to the half-maximal absorbance in the linear section of the dilution curve, or the IC50 value using a non-linear four parameter regression. Normalized titer was calculated by dividing the titer by that of the 2B1.5 clone Ab standard for a given plate. Color coded based on treatment group: black–PBS, red–Blank MP, blue–TRI MP. Spearman correlation coefficient and p value for correlation are indicated. **B)** Average normalized anti-CII IgG 2a Ab titer by treatment group. n = 12 mice per group, data presented as mean ± SEM.
(TIF)

S5 Fig Analysis of LAP, CTLA-4, and CD73 expression by treatment group. Quantification of the percentage of the indicated CD4^+^ T cell population that are LAP^+^, CTLA-4^+^,or CD73^+^ relative to isotype control. Complimentary analysis to Fig 5, but presented by treatment group (PBS, Blank MP, or TRI MP). Graphs are for the FoxP3^-^CD25^+^ population in the iLN (**A-C**), the FoxP3^-^CD25^+^ population in the spleen (**D-F**), the FoxP3^+^CD25^+^ population in the iLN (**G-I**), or the FoxP3^+^CD25^+^ population in the spleen (**J-L**).**)**. n = 6 mice per group, data presented as mean ± SEM, and the following cutoffs were used for significance: ** p < 0.01, *** p < 0.001, **** p < 0.0001.
(TIF)

S6 Fig Lack of appreciable Tbet+ population in the iLN at Day 15 staining with two different antibody clones. Representative flow plots showing CD4 expression versus Tbet expression showing isotype control or 3 different samples each for PBS, Blank MP, and TRI MP treatments (grouped by column). A) Samples stained with Tbet antibody clone 4B10. B) Samples stained with Tbet antibody clone O4-06.
(TIF)

S7 Fig Additional analysis of paw immune infiltrate. **A,C,D-H)** Indicated parameter of the paw immune infiltrate by treatment group (Day 40–42). In two of these (E and F), mice that were not immunized with bCII or treated in any other way are included as an additional control. n = 6–12 mice per group (n = 3 un-immunized), data presented as mean ± SEM. These include the number of CD4^+^ T cells **(A)**, the percentage of CD4^+^ T cells that are FoxP3^+^ **(C)**, the number of CD45^+^ immune cells **(D)**, the percentage of CD45^+^ cells that are monocytes/macrophages (CD11b^+^Ly-6G^-^Ly-6C^+^) **(E)**, the percentage of CD45^+^ cells that are neutrophils (CD11b^+^Ly-6G^+^) **(F)**, the number of TNF-α expressing monocytes/macrophages **(G)**, and the number of TNF-α expressing neutrophils **(H)**. **B)** Percentage of CD4^+^ T cells that are FoxP3^+^ versus arthritis score. Spearman correlation coefficient and p value for correlation are indicated. n = 12 mice per group.
(TIF)
