# Supplementary figures and images for: TRI microparticles prevent inflammatory arthritis in a collagen-induced arthritis model
Source: PLoS One. 2020 Sep 23;15(9):e0239396. doi: 10.1371/journal.pone.0239396 (PMC7510963; doi:10.1371/journal.pone.0239396)

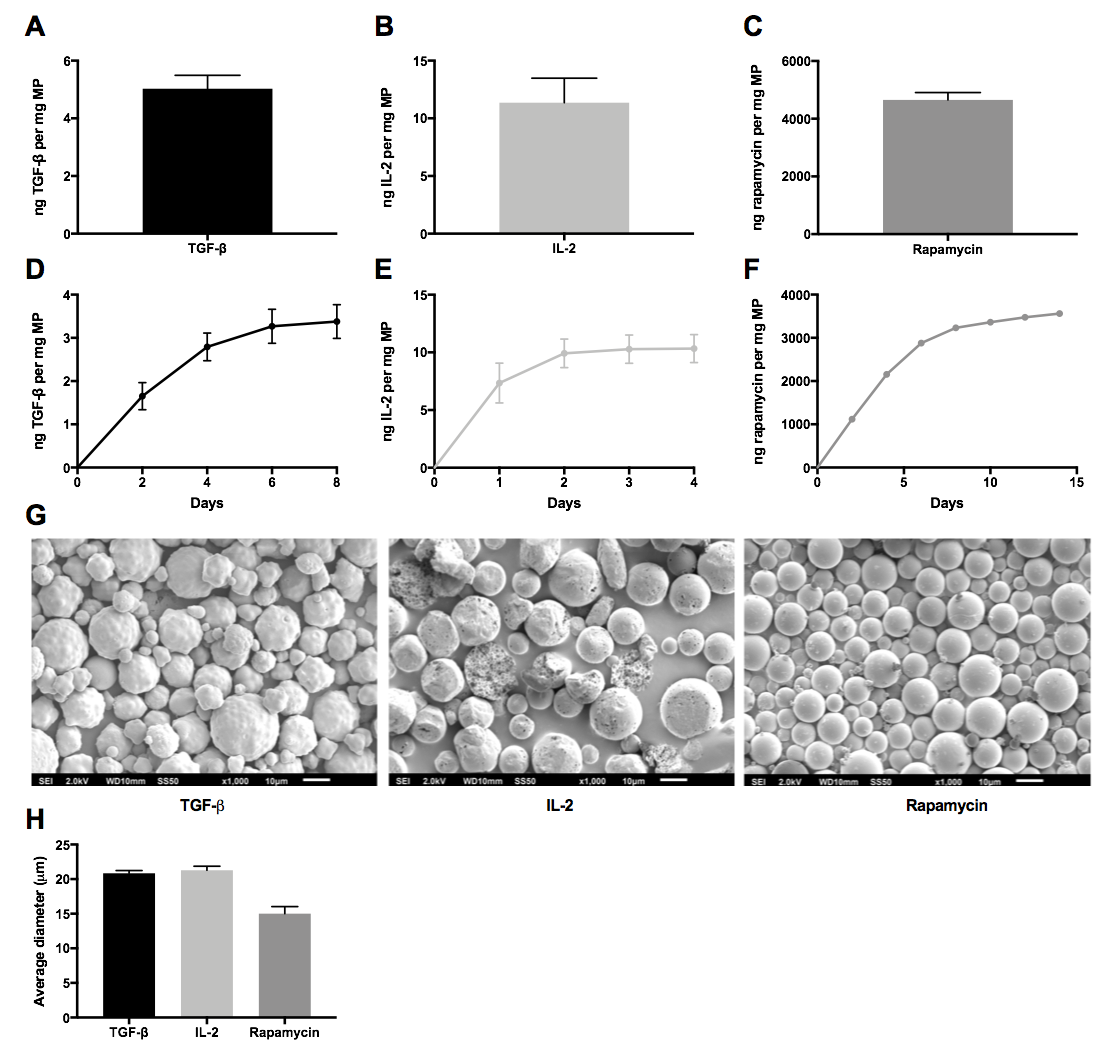

Supplement: S1 Fig — A-C) Drug loading (ng/mg) for TGF-β microparticles (MP) (A), IL-2 MP (B), and rapamycin MP (C) respectively. n = 6–12 batches of MP per group, data presented as mean ± SEM. D-F) In vitro release kinetics for TGF-β MP (D), IL-2 MP (E), and rapamycin MP (F) respectively. Representative batch of MP shown with release samples performed in triplicate and presented as mean ± SEM. G) SEM images showing surface morphology of TGF-β MP, IL-2 MP, and rapamycin MP with 10 μm scale bar shown for reference. H) Average MP diameter measured by Coulter Counter, presented as mean ± SEM. (TIF) [file pone.0239396.s001.tif]

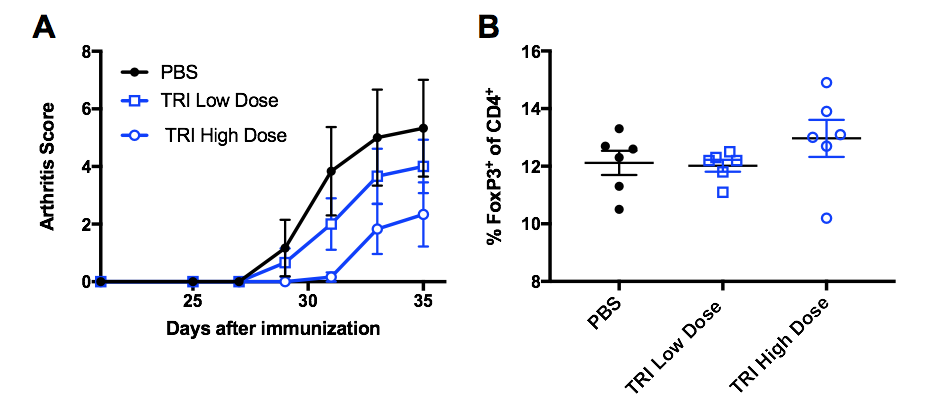

Supplement: S2 Fig — A) Arthritis scores over time for mice given daily injections (Day 0–13) on each flank above the hind limb with 100 μL of PBS, TRI Low Dose (2 ng TGF-β, 1 μg rapamycin, and 2 ng IL-2), or TRI High Dose (20 ng TGF-β, 10 μg rapamycin, and 20 ng IL-2. B) Quantification of the percentage of CD4+ T cells that are FoxP3+ in the draining (inguinal) lymph node on Day 35. n = 6 mice per group, data presented as mean ± SEM. (TIF) [file pone.0239396.s002.tif]

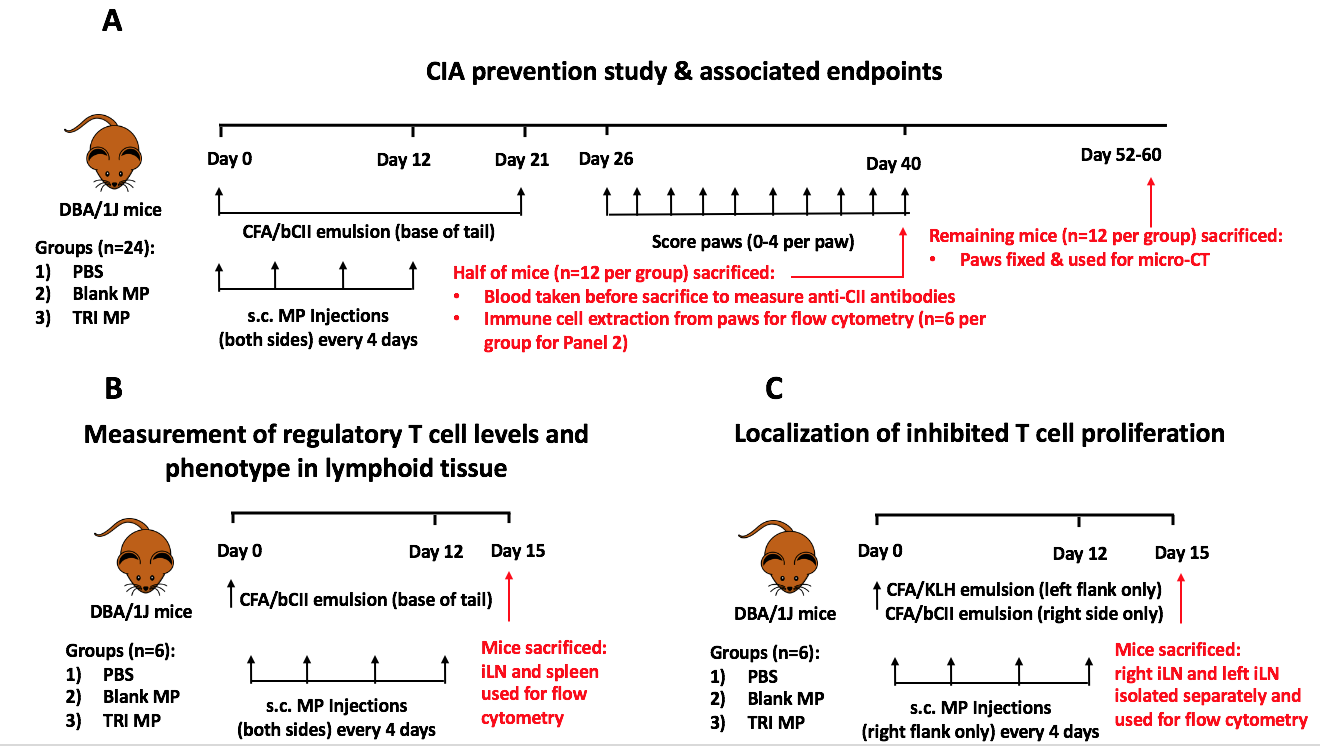

Supplement: S3 Fig — A) Timeline for CIA prevention and associated endpoints. Mice (n = 24 per group) were immunized with an emulsion of complete Freund’s adjuvant (CFA) and bovine collagen II (bCII) at the base of the tail on Day 0 and subcutaneously (s.c.) injected with PBS or microparticles (MP) by both hind limbs every 4 days between Day 0 and Day 12. Mice were scored by a blinded individual for signs of arthritis between Day 26 and Day 40 (Fig 1), at which point half of mice were sacrificed and used to measure serum auto-antibodies (Fig 3) and to extract immune cells from the paws (Fig 6). The other half of mice were left until Day 52–60 to allow sufficient time for inflammation to result in bone erosion, and then sacrificed and used for micro-computed tomography (CT) (Fig 2). B) Timeline for measurement of regulatory T cell levels and phenotype in lymphoid tissue. Mice (n = 6 per group) treated as in A), but sacrificed at Day 15 to assess T cells at a time point close to MP administration to assess regulatory T cell levels and phenotype in the draining inguinal lymph nodes (iLN) and spleen (Fig 4). C) Timeline for localization of inhibited T cell proliferation. Mice (n = 6 per group) were immunized with bCII by the base of the tail on the right side only, and on the left flank an emulsion of CFA and Keyhole limpet hemocyanin (KLH) was given. Mice were treated with PBS or MP as described above, but only on the right flank. T cell responses were assessed for both the draining iLN (right side) and contralateral iLN (left side) relative to MP localization (Fig 5). (TIF) [file pone.0239396.s003.tif]

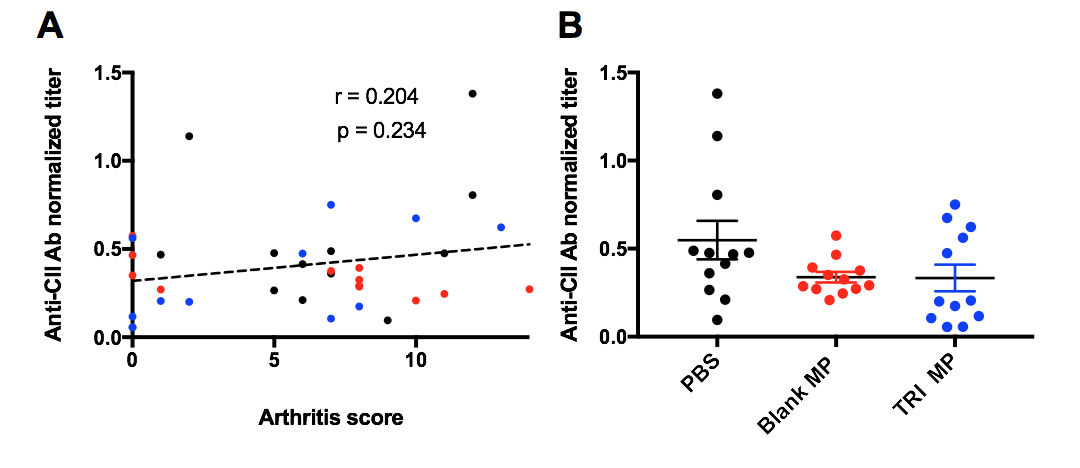

Supplement: S4 Fig — A) Normalized anti-CII IgG2a Ab titer versus arthritis score. Ab titer was defined as the dilution corresponding to the half-maximal absorbance in the linear section of the dilution curve, or the IC50 value using a non-linear four parameter regression. Normalized titer was calculated by dividing the titer by that of the 2B1.5 clone Ab standard for a given plate. Color coded based on treatment group: black–PBS, red–Blank MP, blue–TRI MP. Spearman correlation coefficient and p value for correlation are indicated. B) Average normalized anti-CII IgG 2a Ab titer by treatment group. n = 12 mice per group, data presented as mean ± SEM. (TIF) [file pone.0239396.s004.tif]

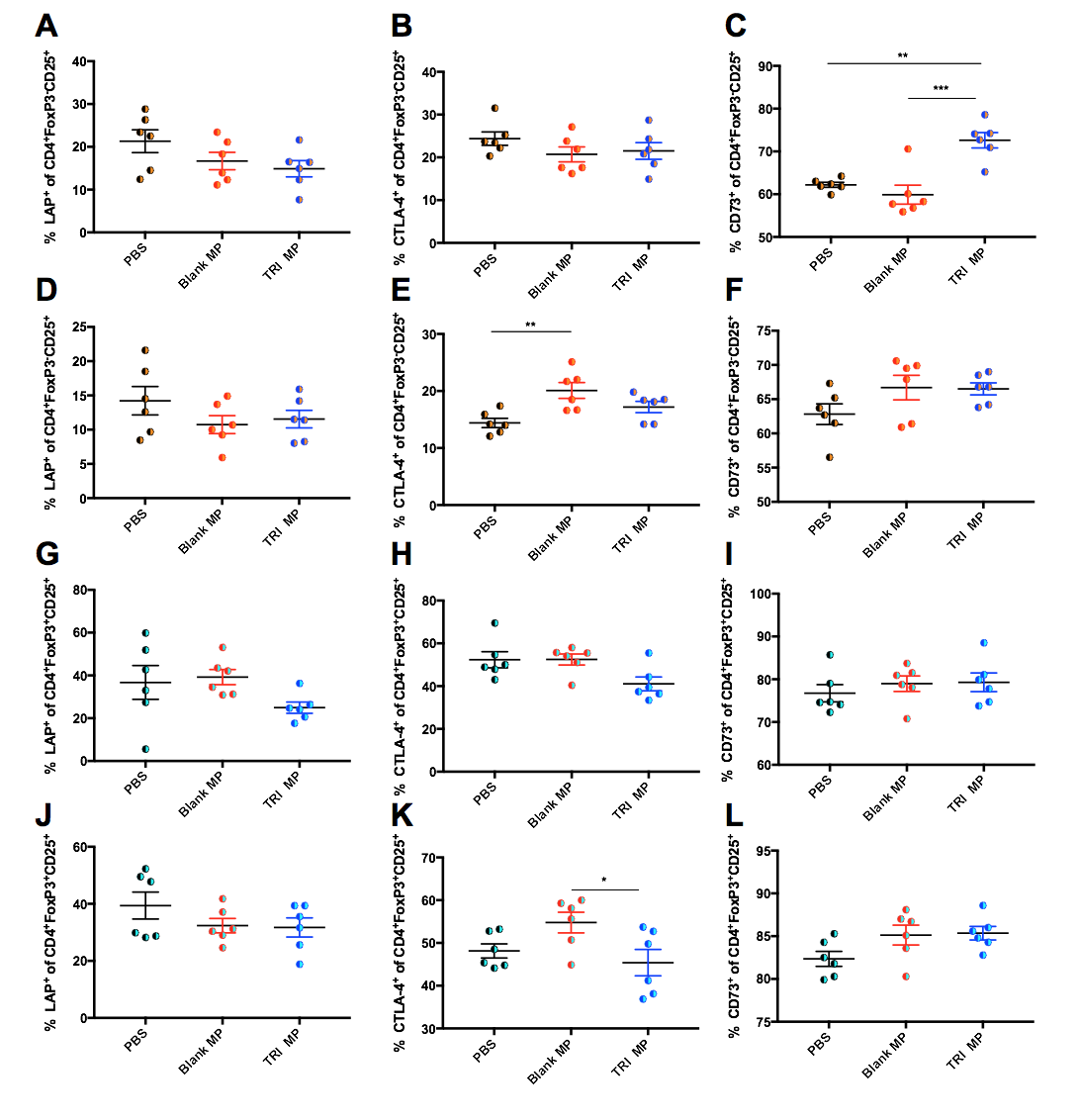

Supplement: S5 Fig — Quantification of the percentage of the indicated CD4+ T cell population that are LAP+, CTLA-4+,or CD73+ relative to isotype control. Complimentary analysis to Fig 5, but presented by treatment group (PBS, Blank MP, or TRI MP). Graphs are for the FoxP3-CD25+ population in the iLN (A-C), the FoxP3-CD25+ population in the spleen (D-F), the FoxP3+CD25+ population in the iLN (G-I), or the FoxP3+CD25+ population in the spleen (J-L).). n = 6 mice per group, data presented as mean ± SEM, and the following cutoffs were used for significance: ** p < 0.01, *** p < 0.001, **** p < 0.0001. (TIF) [file pone.0239396.s005.tif]

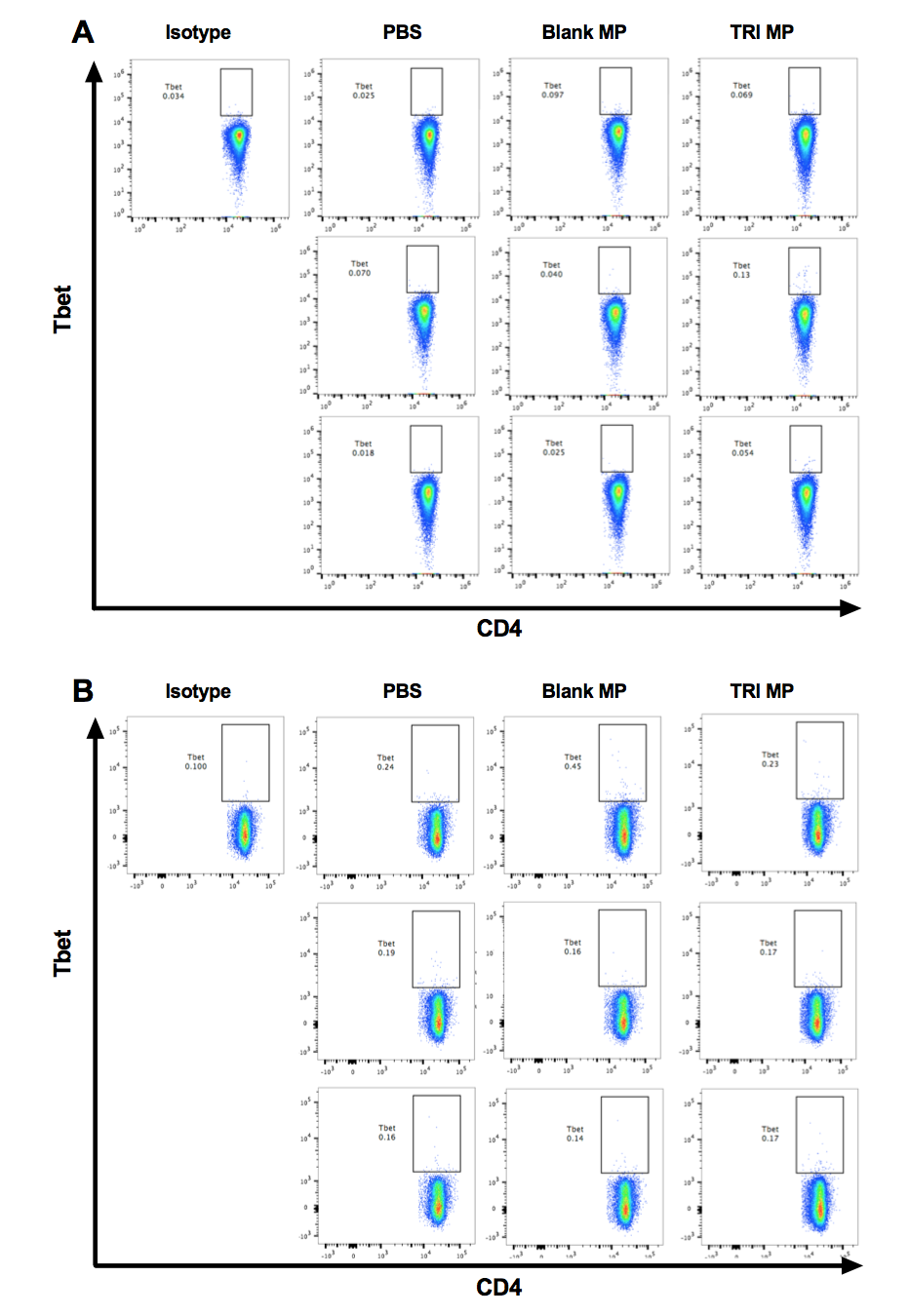

Supplement: S6 Fig — Representative flow plots showing CD4 expression versus Tbet expression showing isotype control or 3 different samples each for PBS, Blank MP, and TRI MP treatments (grouped by column). A) Samples stained with Tbet antibody clone 4B10. B) Samples stained with Tbet antibody clone O4-06. (TIF) [file pone.0239396.s006.tif]

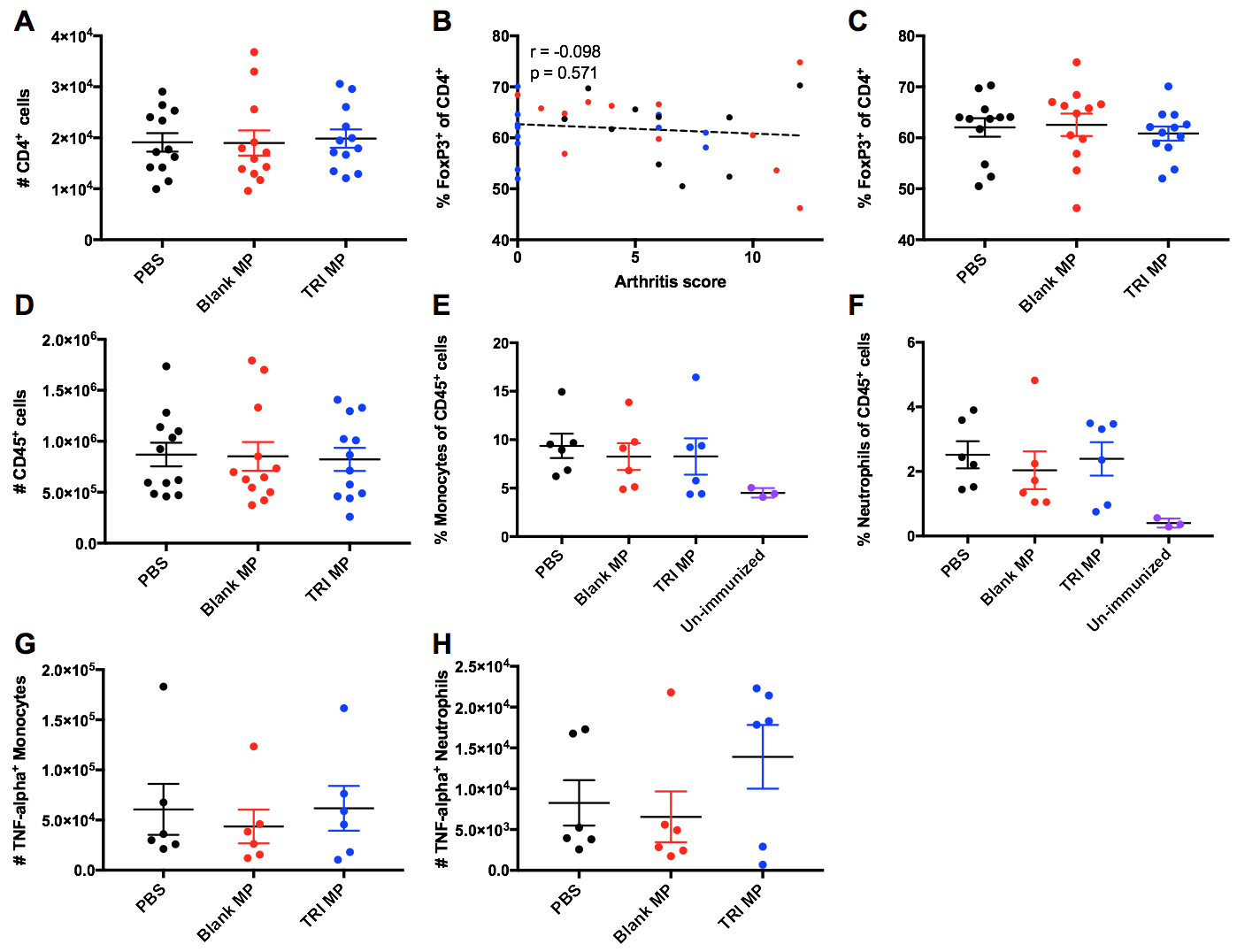

Supplement: S7 Fig — A,C,D-H) Indicated parameter of the paw immune infiltrate by treatment group (Day 40–42). In two of these (E and F), mice that were not immunized with bCII or treated in any other way are included as an additional control. n = 6–12 mice per group (n = 3 un-immunized), data presented as mean ± SEM. These include the number of CD4+ T cells (A), the percentage of CD4+ T cells that are FoxP3+ (C), the number of CD45+ immune cells (D), the percentage of CD45+ cells that are monocytes/macrophages (CD11b+Ly-6G-Ly-6C+) (E), the percentage of CD45+ cells that are neutrophils (CD11b+Ly-6G+) (F), the number of TNF-α expressing monocytes/macrophages (G), and the number of TNF-α expressing neutrophils (H). B) Percentage of CD4+ T cells that are FoxP3+ versus arthritis score. Spearman correlation coefficient and p value for correlation are indicated. n = 12 mice per group. (TIF) [file pone.0239396.s007.tif]
